# Draft Genome Sequence of *Phomopsis longicolla* Type Strain TWH P74, a Fungus Causing Phomopsis Seed Decay in Soybean

**DOI:** 10.1128/genomeA.00010-15

**Published:** 2015-02-19

**Authors:** Shuxian Li, Qijian Song, Pingsheng Ji, Perry Cregan

**Affiliations:** a United States Department of Agriculture, Agricultural Research Service (USDA-ARS), Crop Genetics Research Unit, Stoneville, Mississippi, USA; b USDA-ARS, Soybean Genomics and Improvement Laboratory, Beltsville Agriculture Research Center, Beltsville, Maryland, USA; c Department of Plant Pathology, University of Georgia, Tifton, Georgia, USA

## Abstract

*Phomopsis longicolla* is the primary cause of Phomopsis seed decay in soybean. Here, we report the *de novo* assembled draft genome sequence of the P. longicolla type strain TWH P74 (ATCC 60325), which was originally isolated by Hobbs et al. from soybean seed in Ohio in 1983.

## GENOME ANNOUNCEMENT

*Phomopsis longicolla* T. W. Hobbs is one of the important seed-borne fungal pathogens in the *Diaporthe-Phomopsis* complex and the primary cause of Phomopsis seed decay (PSD) in soybean (1, 2). This disease severely decreases the quality of soybean seeds and the yield (3, 4). Most research has been focused on breeding for resistant lines (5, 6). However, the mechanisms of PSD disease development and pathogen invasion of soybean are not fully understood. As a first step to investigate the genetic base of fungal virulence factors and understand the mechanism of infection, we have assembled the draft genome sequence of P. longicolla type strain TWH P74 (ATCC 60325), which was originally isolated by Hobbs et al. from soybean seed in Ohio in 1983 (1).

Genomic DNA of type strain TWH P74 was extracted from a 4-day-old culture and used to generate mate-pair and paired-end libraries with insert sizes of approximately 4 kb and 500 bp, respectively. A no-gel mate-pair library was generated with the Nextera mate-pair sample preparation kit (Illumina, San Diego, CA), and a paired-end library was made using the TruSeq DNA PCR-free sample preparation kit (Illumina, San Diego, CA) according to the manufacturer’s protocols. Libraries were sequenced in separate lanes on an Illumina HiSeq 2500 sequencer using a TruSeq SBS sequencing kit (version 3, Illumina) at the Genomics Core Facility, Purdue University.

The mate-pair library produced 43,688,054 reads (read length = 101 bp). The paired-end library produced 71,713,088 reads (read length = 101 bp). After removing the adapter sequences and trimming poor quality bases (Phred score < 20) for each read using Trimmomatic (7) and/or fastx clipper (http://hannonlab.cshl.edu/fastx_toolkit/) and eliminating reads below 30 bases, a total of 29,788,854 mate-pair reads (2,554 Mb) and 68,445,472 paired-end reads (6,748 Mb) were retained. A draft of the P. longicolla genome was assembled from both libraries using software ABySS *de novo* assembler version 1.3.7 at a k-mer of 90 (8). The assembly contained a total of 986 scaffolds with average read depth coverage of 145-fold. The *N*_50_ was 213.1 kb, and the maximum contig length was 1,124 kb. The total sequence length of the resulting draft genome was 64,714,586 bp with an overall G+C content of 48.1%. Gene annotation using the Augustus program (http://bioinf.uni-greifswald.de/webaugustus/prediction/create) (9) trained with the parameters of the species Fusarium graminearum resulted in 16,606 genes. The average length of the gene is 1,709 bp ranging from 215 bp to 23,099 bp. The total length of the gene sequence was 28.4 Mb, which is 43.8% of the whole-genome sequence. We used CEGMA (10) against a set of 248 conserved protein families that occur in a wide range of core eukaryotic gene datasets (CEGs) (http://korflab.ucdavis.edu/Datasets/genome_completeness/index.html#SCT2) and 98.21% of the core genes were matched, indicating the draft genome sequence was substantially complete.

### **Nucleotide sequence accession number**s.

The draft genome sequence of P. longicolla type strain TWH P74 has been deposited at DDBJ/EMBL/GenBank under the accession no. JUJX00000000. The version described in this paper is version JUJX01000000.
